# Stable isotope records of sei whale baleens from Chilean Patagonia as archives for feeding and migration behavior

**DOI:** 10.1002/ece3.5939

**Published:** 2019-12-18

**Authors:** Lilian Reiss, Verena Häussermann, Christoph Mayr

**Affiliations:** ^1^ Institute of Geography Friedrich‐Alexander‐Universität Erlangen‐Nürnberg Erlangen Germany; ^2^ Huinay Scientific Field Station Escuela de Ciencias del Mar Facultad de Recursos Naturales Pontificia Universidad Católica de Valparaíso Valparaíso Chile; ^3^ GeoBio‐Center Department of Earth and Environmental Sciences Ludwig‐Maximilians‐Universität München Germany

**Keywords:** *Balaenoptera borealis*, baleen growth rate, carbon isotopes, mass mortality event, nitrogen isotopes, Patagonian fjords

## Abstract

Carbon (δ^13^C) and nitrogen (δ^15^N) stable isotope variations in baleen plates of sei whales (*Balaenoptera borealis*) stranded after a mass mortality event in Chilean Patagonia were investigated to assess potential dietary and migratory patterns. Carbon and nitrogen isotope ratios of seven baleens from six individuals were analyzed. The δ^13^C values ranged from − 19.1 to − 15.9‰ and the δ^15^N values from 8.7 to 15.4‰. Variations of up to 2.9‰ for δ^13^C and 5.3‰ for δ^15^N were observed within one baleen. Carbon and nitrogen isotope records of each baleen were significantly correlated and showed recurring oscillations confirmed by wavelet analyses. Oscillations slightly differed in periodicity indicating variable baleen growth rates between 10.0 and 16.5 cm/year. Food sources of the whales are discussed in terms of available isotope data for potential prey taxa and potential migratory behavior on the basis of latitudinal isotope gradients of particulate organic matter. Cyclicity could be explained by regular migrations of the sei whales from subtropical calving areas to high‐latitude foraging grounds. δ^15^N records of baleens differed between individuals eventually pointing to diverse feeding and migratory preferences among sei whale individuals.

## Graphical abstract

We analyzed carbon and nitrogen stable isotopes of southern sei whale baleens from a mass mortality event in Chilean Patagonia to assess dietary and migratory patterns. Oscillating isotope patterns of baleens were statistically analyzed and related to seasonal latitudinal migrations. Different feeding habits and migration routes are evident in the baleen isotopic records.

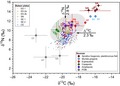

## 1 INTRODUCTION

The largest historically documented baleen whale mass mortality event (MME) took place in the Patagonian fjord area (Chile) between February and April 2015. At least 343 stranded balaenopterids were recorded within these months, many during aerial observations. From these, 29 individuals were accessed by boat and could be taxonomically identified as sei whales (*Balaenoptera borealis* lesson, 1828) (Häussermann et al., 2017). Most of the whales stranded in two assemblages of altogether 298 individuals found in an area of only 0.87 km^2^ (Häussermann et al., 2017). MMEs are uncommon for baleen whales (Geraci et al., 1989; Rowntree et al., 2013), since these whales tend to be solitary or form only small groups (Bannister, 2009). MMEs of baleen whales were reported only rarely before, the largest one in a restricted period included 14 individuals (Geraci et al., 1989). The MME of 2015 was most likely linked to the presence of a harmful algal bloom (HAB) that was detected in the area in March. The HAB likely caused paralytic poisoning in various marine animal taxa, including the whales (Häussermann et al., 2017). It was triggered by an El Niño anomaly in the years 2014 − 16, which was among the strongest recorded (Newman, Wittenberg, Cheng, Compo, & Smith, 2018).

The stranded whales offered the opportunity to sample baleens to investigate their life habits using isotope geochemical techniques. Such analyses could provide information about the so‐far understudied whale populations in the Southern Hemisphere. In particular, life habits and migratory behavior of southern hemispheric sei whales are not well known (Horwood, 1987; Olsen et al., 2009). The subpolar and polar regions are frequently considered as main feeding areas of baleen whales in general, because of high food accumulations there (Lockyer, 1981). Baleen whales therefore undertake extensive seasonal migrations, ranging from summer feeding grounds in high‐latitude waters to winter calving grounds in low‐latitude waters (Bannister, 2009; Kawamura, 1980). It is assumed that sei whales migrate in a slightly smaller latitudinal range than other balaenopterids, comprising more temperate waters (Horwood, 1987, 2009; Mizroch, Rice, & Breiwick, 1984). The seasonal movements of sei whales in the Southern Hemisphere may extend from the subtropical convergence in the north to the Antarctic convergence in the south (Horwood, 1987).

Extensive migrations of baleen whales, associated with seasonal feeding and potential fasting periods, are reflected on the surface of baleen plates, where they leave a series of visible growth lines (Rice, 2009). Moreover, seasonal movements are recorded in the isotopic signature of continuously growing, metabolically inert animal tissues, such as keratin‐based baleens (Rubenstein & Hobson, 2004). Their nitrogen and carbon isotope values reflect the isotopic composition of the animal's diet shortly before tissue formation transformed by isotopic fractionation. The isotopic composition of an animal's diet in turn is determined by biogeochemical processes, which can vary among geographical regions. Such differences are reflected throughout the food web in organic tissues (DeNiro & Epstein, 1978; Hobson, 1999; Tomaszewicz, Seminoff, Ramirez, & Kurle, 2015), including baleen plates (Best & Schell, 1996). This could be of use for investigating provenance and migratory patterns of animals using carbon and nitrogen stable isotopes. Additionally, metabolic isotope fractionations modify the isotopic composition of an animal relative to its diet. Isotopic fractionation (Δ) is defined as the difference in the isotope ratio (e.g., ^15^N/^14^N or ^13^C/^12^C) between a source (in this case diet) and a product (in this case animal tissue) (Peterson & Fry, 1987). In many cases, isotope fractionation leads to an enrichment of one isotope over the other in the product. While carbon stable isotopes show comparatively little heavy‐isotope enrichment of animals relative to their food, stable nitrogen isotopes increase by 1 to 5‰ (average about 3.4‰ for aquatic food webs; Post, 2002) per trophic level (Peterson & Fry, 1987). Hence, stable isotope analysis is also valuable for tracing trophic relationships between organisms (Best & Schell, 1996; Busquets‐Vass et al., 2017; Fry, 2006; Hobson, 2008; Newsome, Clementz, & Koch, 2010; Post, 2002). However, the trophic heavy‐isotope enrichment also depends on taxon, excretion type of an organism, feeding habits, and the analyzed tissue type (DeNiro & Epstein, 1978; Peterson & Fry, 1987; Schoeninger & DeNiro, 1984; Vanderklift & Ponsard, 2003).

In addition to isotope fractionation, the isotopic values of an animal are also potentially influenced by isotopic variability of their food on temporal and spatial scales. Attempts to capture geographical isotope variability in the marine realm are done by modeling baseline isotope values of food webs using so‐called isoscapes (Jaeger, Lecomte, Weimerskirch, Richard, & Cherel, 2010). However, the database of such isoscapes is often insufficient, which is especially the case for our study area, the southeastern Pacific (Trueman and St. John Glew, 2019).

Considering their food uptake over wide‐ranging geographical regions, baleen whales were frequently studied using stable isotope analyses. Isotopic oscillations of their diet are especially well reflected in the metabolically inert baleen plates (Best & Schell, 1996; Busquets‐Vass et al., 2017; Hobson, 1999). The baleen plates are composed of calcified keratin (Szewciw, De Kerckhove, Grime, & Fudge, 2010) and continuously grow during the lifespan of a baleen whale (Rice, 2009; Szewciw et al., 2010), thus giving insights into feeding habits and seasonal migration. Additionally, physiological parameters like fasting (Hobson, Alisauskas, & Clark, 1993), age and sex (Mendes, Newton, Reid, Frantzis, & Pierce, 2007), or pregnancy (Borrell et al., 2013) could have an influence on whale tissue isotope ratios. For instance, fasting leads to a ^15^N enrichment in animal tissues due to the preferential excretion of the lighter ^14^N during metabolic activity (Ambrose, 1991) and concomitant ^15^N enrichment during catabolic reactions (Hobson et al., 1993).

The main research questions of this study were to use isotope patterns in southern sei whale baleen plates to investigate (1) the contributions of prey items to their diet and (2) elucidate whether seasonal movement patterns are observable. Stable carbon and nitrogen isotope ratios of baleen plates were analyzed to address these questions.

## 2 MATERIAL AND METHODS

### 2.1 Sampling

**Figure 1:**
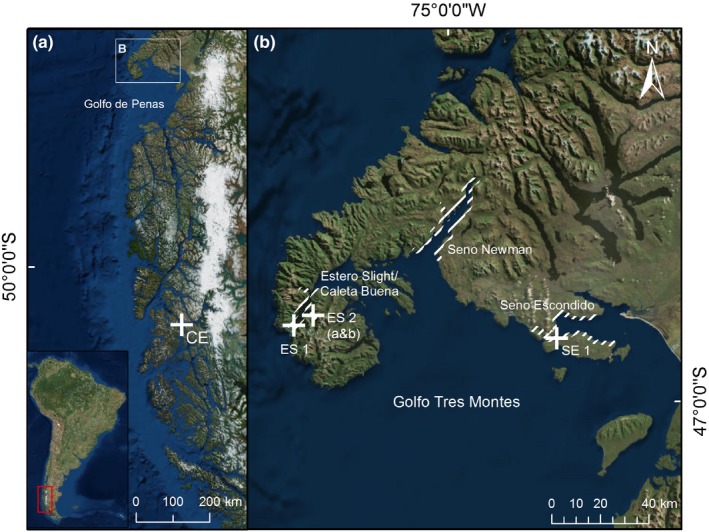
Location of the different sampling sites. (a) Overview of the research area in the Patagonian fjord region. (b) Detailed view of Golfo Tres Montes and the main sampling sites Seno Escondido (SE), Seno Newman (SN), and Estero Slight/Caleta Buena (ES). The shaded areas indicate the regions where samples were taken. Exact coordinates are missing for SN and SE 2. (Source: Service Layer Credits: ESRI, HERE, Garmin, © OpenStreetMap contributors, and the GIS user community)

All baleens investigated in this study were taken from sei whales. The majority of the dead whales was sampled in the year 2016 during several expeditions to the Central Patagonian zone between 46 and 48°S around Golfo Tres Montes. Only one baleen (sample ID CE in Table 1) was sampled further south (Figure 1a), during a previous expedition in May 2015. The sampling sites were mainly along the coasts of Seno Escondido (SE), Seno Newman (SN), and Estero Slight/Caleta Buena (ES), which are located within Golfo Tres Montes, the northern part of Golfo de Penas (Figure 1b) (Häussermann et al., 2017). Due to the remoteness of the area and the already strongly decomposed carcasses, no information about sex, and only imprecise information about size and age is available. With regard to size, all individuals were adults except individual ES2 (Table 1). All baleens were removed manually or with knives taking care of obtaining complete baleens including the part embedded in the gum. The baleens were sampled with Sernapesca sampling permit no. 2016‐11‐10 and shipped to Germany with CITES permit no. 16CL000004WS for further analysis.

**Table 1:** Details of sei whales from which baleen plates were available

Sample ID	Site	Sampling date	Geographical coordinates	Stage of life	No. of baleen plates	Total baleen length (cm)
ES 1 (*371*)	Estero Slight/Caleta Buena	16 Feb 2016	46°48.525'S 75°34.157'W	Adult	1	40.5
ES 2 (a&b) (*377*)	Estero Slight/Caleta Buena	23 Feb 2016	46°47.072'S 75°29.847'W	Juvenile	2	a: 25.0 b: 28.5
SN	Seno Newman	Feb 2016	***	Adult	1	59.0
SE 1 (*90*)	Seno Escondido (San Quintin Bay I)	Feb 2016	46°50.437'S 74°35.943'W	Adult	1	59.0
SE 2	Seno Escondido	Feb 2016	***	Adult	1	26.0
CE (*332*)	Canal Esteban (Paso Isaza)	14 May 2015	50º53.983'S 74º18.133'W	Adult	1	41.0

Note

Italic numbers in brackets after sample IDs correspond to whale IDs in Häussermann et al. (2017), if available. All whales stranded, except for CE, which carcass was hit by a “Navimag” ferry. Date and site indicate when and where the baleen plates were sampled. For fields with the symbol ***, information is missing.

### 2.2 Baleen plates processing

Baleen plates from six sei whale individuals were available; two baleens (ES 2 a&b) stem from the same individual while all other individuals are represented only by one baleen. Baleen plates were sampled consecutively by cutting 0.5‐cm‐wide pieces from the basis to the end along the outside edge with a hand saw. Each sample was then clipped off with pliers. The samples were stored in 2.0‐ml Eppendorf tubes and soaked in distilled water for four hours. To remove potential impurities, each sample was then treated for ten seconds in deionized water in an ultrasonic bath (Sonorex, Bandelin, Germany). Afterward, samples were frozen and lyophilized for 75 hr. For homogenization, each sample was ground using a centrifugal mill (Pulverisette 14, Fritsch, Germany) and again stored in Eppendorf tubes. We tested a pretreatment with lipid extraction according to Borrell, Abad‐Oliva, Gómez‐Campos, Giménez, and Aguilar (2012) for a subset of ten samples. As we found no significant difference between ultrasonic treatment and lipid‐extracted samples in both δ^15^N and δ^13^C, we refrained from a lipid extraction procedure.

### 2.3 Stable isotope analysis

Between 270 and 290 g of each powdered sample (dried baleen) was weighed into tin capsules using a microbalance (Sartorius, ME36S, Germany). For stable isotope analysis, an elemental analyzer (EA) (NC2500, Carlo Erba, Italy) coupled to an isotope ratio‐mass spectrometer (IRMS) (DeltaPlus, Thermo‐Finnigan, Germany) was used. All results are expressed as delta (δ) values in per mil (‰) relative to international standards.

The δ notation is defined as:

(1) $$\delta =(R_{\text{Sample}}/R_{\text{Standard}}-1)$$

where R is the mass ratio of the heavier isotope to the lighter isotope (^13^C/^12^C or ^15^N/^14^N) of the sample and an international standard, Vienna‐Pee Dee Belemnite (V‐PDB) for carbon and atmospheric N_2_ (AIR) for nitrogen, respectively (Coplen, 2011).

Stable isotope ratios were calibrated using the international standards IAEA CH7 for carbon, IAEA N1 and IAEA N2 for nitrogen. Additionally, a laboratory‐internal organic isotope standard (Peptone‐II) and USGS 41 were used for both carbon and nitrogen isotope calibration. Elemental standards (cyclohexanone‐2,4‐dinitrophenylhydrazone C_12_H_14_N_4_O_4_ and atropine C_17_H_23_NO_3_) were used to infer weight percentages of carbon (%) and nitrogen (%) from peak areas of the chromatograms. The element concentrations were used to calculate molar C/N ratios.

The precision was always ≤0.2‰ (1 standard deviation) for both δ^13^C and δ^15^N. The analytical error of the element concentrations was less than 5%.

### 2.4 Statistical analyses

Shapiro–Wilk tests were applied to test for the normal distribution of data. Spearman's rank‐correlation coefficients were used to check for relationships between δ^15^N and δ^13^C within a single baleen plate. Statistical calculations were carried out with R Studio (version 3.4.3 and 3.4.4).

For wavelet analyses, the dendrochronology library in R (dlpR package; Bunn et al., 2019) was implemented. The significance of frequencies was evaluated using the Morlet wavelet tool within the package. In general, wavelet analysis is used to detect significant frequencies within a time series. The isotopic sequences of serially sampled baleens can be treated as time series, as baleens grow continuously throughout the life of a baleen whale (Rice, 2009) and samples were taken at defined intervals of 0.5 cm distance. This method can further be used to detect transitions of dominant periods within a time series. For all tests performed, a significance level of 0.05 or higher was chosen.

The stable isotope mixing model of Phillips and Koch (2002) was used to estimate percentages of different food sources.

## 3 RESULTS

### 3.1 Isotopic variability in baleen plates

**Figure 2:**
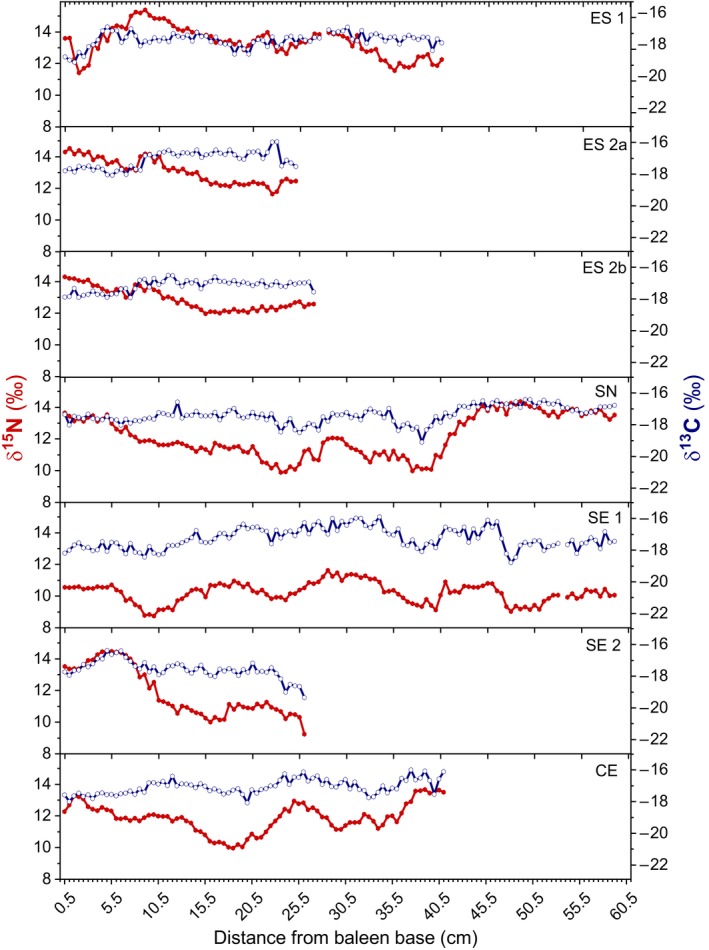
δ^15^N (solid, red) and δ^13^C (open, blue) values of the seven baleen plates

Average δ^15^N values of the baleens were between 10.2‰ (SE 1) and 13.4‰ (ES 1), and δ^13^C between −17.1‰ (ES 2a, CE) and −17.6‰ (SE 2) (Figure 2). The mean δ^15^N range of all baleens was 3.6‰, and the mean δ^13^C range was 2.3‰. The baleen plate SE 2 exhibited a particularly large range of 5.3 ‰ in the δ^15^N values. Similarly, this baleen plate showed the highest variation of 3‰ in the δ^13^C values. The baleen SN also showed a remarkably high variability of 4.5‰ within the δ^15^N value range followed by ES 1 (4.0‰) and CE (3.7‰). δ^15^N values of the baleens from the only juvenile individual from our dataset (ES 2a&b) showed less variability than all the others. The lowest δ^15^N values (down to 8.7‰) were found in baleen SE 1. The N and C isotope ratios of all baleens were significantly positively correlated except of one individual (ES 2), which was significantly negatively correlated (ES 2a: *r*_s_ = −0.52, *p* < .0001, *n* = 50; ES 2b: *r*_s_ = −0.51, *p* < .0001, *n* = 54). The highest correlation between δ^15^N and δ^13^C was reached for the baleens SN (*r*_s_ = 0.67, *p* < .0001, *n* = 118), SE 1 (*r*_s_ = 0.67, *p* < .0001, *n* = 118), and SE 2 (*r*_s_ = 0.67, *p* < .0001, *n* = 52). Only a weak correlation existed between δ^15^N and δ^13^C for the baleens ES 1 and CE (ES 1: *r*_s_ = 0.27, *p* < .05, *n* = 82; CE: r_s_ = 0.23, *p* < .05, *n* = 82). The nitrogen and carbon contents of the different baleens were rather uniform, with a molar C/N ratio of 3.9 ± 0.15 (1 standard deviation) on average.

### 3.2 Baleen growth determination

**Figure 3:**
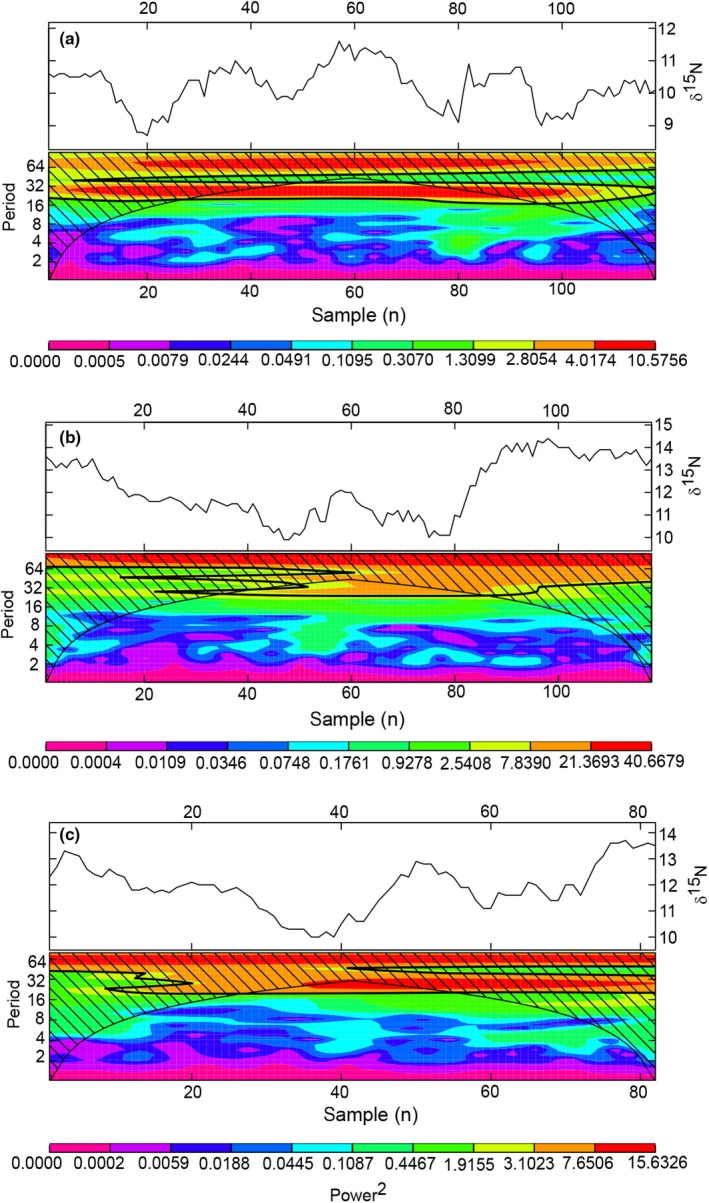
Significance of frequencies of oscillations in δ^15^N values of the baleens “SE 1” (a), “SN” (b), and “CE” (c). The upper panel in each graph shows the δ^15^N values of the different baleens. The lower panel indicates significant frequencies within the δ^15^N values of the baleen, showing also the significant interval, as the x‐axis represents the position of each sample (n). Note, that each sample number represents a consecutive step of 0.5 cm. The bell‐shaped curve represents the cone of influence, that is, the frequency limits. The diagonal lines mark the area outside the cone of influence. The black solid line within the cone of influence indicates the level of significance (*p* < .05). Colors represent the power of the underlying wavelet signal

Oscillations were evident in the δ^15^N, and less pronounced in the δ^13^C values of all baleens (Figure 2). Baleen growth rates were estimated based on tentative seasonal cycles in δ^15^N values and assuming that one cycle represents one year. δ^15^N were preferred over δ^13^C values in the detection of cycles due to their higher fluctuations and generally stronger signal. The results of the wavelet analysis only showed significant frequencies in the three longest baleens (SE 1, SN, and CE), with SE 1 being the only baleen with three significant fully defined cycles (Figure 3a). For the baleen SE 1, a significant range inside the cone of influence could be recognized from sample 27–92 and from sample 46 to 73, which is equivalent to 13.5–46.0 cm and to 23.0 to 36.5 cm (Figure 3a). Considering the baleen plate SN, significant frequencies were detected from sample 37 to 82 (18.5 to 41.0 cm) (Figure 3b), whereas the significant frequencies in CE ranged from sample 27 to 56, that is, from 13.5 to 28.0 cm (Figure 3c). Although only two or three cycles were determined, the persistent frequency suggests that the cycles show a recurring pattern at regular intervals. Since each sample represents 0.5 cm, a period of about 10.0 to 16.5 cm in the baleen SE 1 was calculated by multiplying the significant periods (20 to 33) with the respective sampling interval (0.5 cm). Based on this calculation, a cycle length of 10.0 to 14.0 cm was estimated for CE and SN (significant periods of 20 and 28, respectively), signifying that the average growth rates of the baleen plates were 10.0 to 16.5 cm /year.

## 4 DISCUSSION

### 4.1 Evaluation of potential food sources

We demonstrated, in accordance with previous investigations on a fin whale (García‐Vernet, Sant, Víkingsson, Borrell, & Aguilar, 2018), that baleens from the same individual (ES 2a&b) recorded similar isotopic variations (Figure 2). This is a prerequisite for the interpretation of baleen isotope variations as archive of the individual's life history. A small divergence between the two baleens at 20.0 and 21.5 cm, however, might be related to impurities or compositional differences. Since the keratin of the baleen is a metabolically inert tissue, it approximately reflects the isotopic source value and nutritional status at the location where the tissue was synthesized (Hobson, 1999; Schell, Saupe, & Haubenstock, 1989). Due to the fast turnover rates of keratin, the most recently produced part of a baleen approximately integrates food sources of the last two weeks for different species (Best & Schell, 1996; Caraveo‐Patiño, Hobson, & Soto, 2007; Eisenmann et al., 2016; Matthews & Ferguson, 2015).

Individual feeding differences could explain the overall lower δ^15^N (but not δ^13^C) values of SE 1 (mean 10.2 ± 0.6‰) compared to the other studied sei whales (mean δ^15^N values: 11.9 to 13.4‰). The isotopic composition of the sei whales’ prey can be inferred from the equation (Peterson & Fry, 1987).

(2) $$\Delta =\delta X_{\text{tissue}}-\delta X_{\text{diet}}$$

where Δ = trophic isotope fractionation and δX = δ^15^N or δ^13^C, respectively.

**Figure 4:**
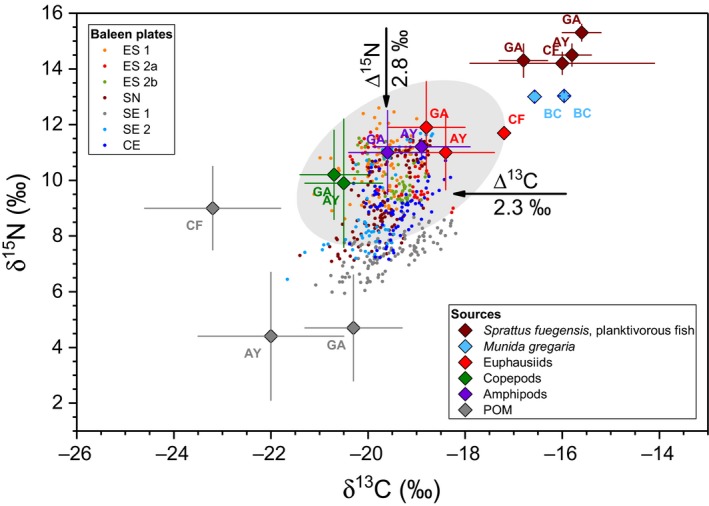
δ^15^N versus δ^13^C values of baleen plates (small filled circles) corrected for trophic fractionation (Δ^13^C and Δ^15^N, respectively, from Borrell et al., 2012). Additionally, values of potential prey items are given: *Sprattus fuegensis* (Montecinos et al., 2016; Sepúlveda et al., 2015), small planktivorous fish (Mayr et al., 2011), *Munida gregaria* (Pérez‐Barros et al., 2010), euphausiids (Mayr et al., unpublished; Montecinos et al., 2016), and copepods (Montecinos et al., 2016). POM values are from Mayr et al. (2011) and Montecinos et al. (2016). The labels correspond to different sampling regions in Patagonia and Tierra del Fuego (AY: Aysén region, BC: Beagle Channel, CF: Comau Fjord, GA: Golfo de Ancud). Bars represent standard deviations of multiple samples. The shaded area represents the presumed field of baleen isotope values if euphausiids, amphipods, and copepods were the main food source. Baleen values outside of the field are not explained by Patagonian food sources

Commonly assumed Δ^15^N and Δ^13^C values in ecological studies are 3.4‰ and 0.4‰, respectively (Post, 2002). However, these values can vary largely depending on tissue type and other factors (Fry, 1988; Vanderklift & Ponsard, 2003). Borrell et al. (2012) suggested a mean Δ^13^C of 2.3 ± 0.3‰ and a mean Δ^15^N of 2.8 ± 0.3‰ for baleen plates of fin whales relative to their preferential food (i.e., krill). These Δ values were corroborated in a subsequent study on humpback whales in the Southern Hemisphere (Eisenmann et al., 2016) and thus were used herein. A δ^13^C_diet_ range of − 21.7 to − 18.2‰ is the result when Equation (2) is applied to the total range of the baleen δ^13^C values. Using a mean Δ^15^N of 2.8 ± 0.3‰ (Borrell et al., 2012) provides a total δ^15^N_diet_ range of 5.9 to 12.6‰ for the sei whale food (Figure 4).

Isotopic studies of Patagonian fjord food webs are scarce, all the more isotopic information about Southern Hemisphere sei whales and their prey. Potential food sources may include crustaceans and planktivorous fish from Patagonian fjords representing the last whereabouts of the investigated sei whales. δ^15^N values around 13‰ and δ^13^C values around − 16‰ were reported for the squat lobster *Munida gregaria* in the Beagle Channel (54°51’S; Pérez‐Barros, Romero, Calcagno, & Lovrich, 2010), which is considered an important prey of Patagonian sei whales (Häussermann, 2017; Lockyer, 1981). Patagonian sprat (*Sprattus fuegensis*), an important forage fish in the Patagonian marine ecosystems, provided δ^15^N values of 14.5 ± 0.4‰ and δ^13^C values of −16.6 ± 0.8‰ in the Aysén region close to where the stranded sei whales were found (44 − 47°S; Montecinos, Castro, & Neira, 2016). Elsewhere in Chilean Patagonia, similar values were recorded for this species (Sepúlveda et al., 2015) and other planktivorous fish (Mayr et al., 2011) (Figure 4). In the Aysén area, euphausiids had δ^15^N values of 11.0 ± 1.5‰, and δ^13^C values of − 18.4 ± 1.0‰ and similar values further north in the Inner Sea of Chiloé, that is, the Golfo de Ancud (δ^15^N = 11.9 ± 1.5‰, δ^13^C = −18.8 ± 0.8‰). Copepods had δ^15^N values of 9.9 ± 2.3‰ and δ^13^C values of − 20.5 ± 0.8 ‰ (Montecinos et al., 2016). A single euphausiid sample from the Comau fjord (42°S) provided a δ^15^N value of 11.7‰ and a δ^13^C value of − 17.2‰ (Mayr, unpublished data). In contrast to potential prey items, particulate organic matter (POM) isotope values from Patagonian fjords are highly variable, possibly related to varying terrestrial influence (Vargas et al., 2011) and dependent on predominant size fraction (Mayr et al., 2011).

δ^15^N and δ^13^C values of potential prey items and POM are summarized together with baleen data in Figure 4. Based on the available dataset, euphausiids, amphipods, and copepods from the Aysén area plot within the field of baleen isotope values and, thus, are the most likely prey items (Figure 4). The regional food signal from Patagonian fjords may only be reflected in the baleen section formed immediately before death. We considered these values (i.e., the basal 5 cm of each baleen) and corrected them for trophic isotope fractionation using the values of Borrell et al. (2012). A δ^15^N value of 10.2‰ and a δ^13^C of − 20.0‰ result from this correction. Using these values and those of the three most likely prey items (according to Figure 4), we calculated the proportions of each prey source with a stable isotope mixing model (Phillips & Koch, 2002). The model suggests a prey mixture of 74% copepods, 18% euphausiids, and 8% amphipods.

Whereas the δ^15^N maximum values are in total agreement with the isotopic signature of Patagonian euphausiids, amphipods, and copepods, none of the potential food sources from the fjord area could explain the minimum values (Figure 4). Therefore, the minimum δ^15^N values point to food uptake at habitats outside of the Patagonian fjord region. Physiological effects due to fasting can be excluded as they would have increased δ^15^N values (Hobson et al., 1993; Hobson & Schell, 1998). In contrast, however, Aguilar, Giménez, Gómez‐Campos, Cardona, and Borrell (2014) suggest that δ^15^N maxima in baleens of fin whales off Galicia are more likely related to times of intensive feeding rather than fasting. This was explained by the fact that baleen whales accumulate high amounts of lipids (blubber) as energetic reserves, which could allow them to sustain catabolism during periods of limited feeding (Aguilar et al., 2014). To sum up, the δ^15^N minimum values in our sei whale baleens so far remain unexplained and the fasting hypothesis is not applicable here.

### 4.2 Possible causes for cyclic variations

Seasonal isotope cycles in the baleens were frequently referred to migratory behavior of whales reflecting latitudinal isotope variations in the baselines of marine food webs (Best & Schell, 1996; Eisenmann et al., 2016; Hobson & Schell, 1998; Schell et al., 1989). Apart from latitudinal migrations, also inshore–offshore movements could be reflected in isotope variations of marine predators (Cherel & Hobson, 2007). Matthews and Ferguson (2015) explained the synchronous δ^13^C and δ^15^N increases in bowhead whale (*Balaena mysticetus*) baleens by migratory behavior and year‐round foraging. Many populations of mysticetes migrate between their calving areas in low‐latitude waters in the winter and their preferred feeding habitats in higher latitudes in the summer which is assumed also for sei whales (Horwood, 2009; Mizroch et al., 1984). Sei whales were observed in a wide area in the southeastern Pacific reaching from west of Juan Fernández Islands (32 − 34°S 79 − 89°W in austral winters; Aguayo, Bernal, Olavarría, Vallejos, & Hucke, 1998) to the Magellan Strait (53 − 54°S 70 − 72°W in austral summers; Acevedo et al., 2017). Globally, δ^13^C values of particulate organic matter (δ^13^C_POM_), representing baseline values of marine food webs, exhibit a large isotopic range dependent on sea surface temperature and latitude (Goericke & Fry, 1994; Magozzi, Yool, Vander Zanden, Wunder, & Trueman, 2017). The largest δ^13^C_POM_ shift from about −16 to −26‰ was observed for latitudes between 40° and 60°S (Goericke & Fry, 1994). This latitudinal band includes the area where the whale carcasses were discovered, and thus, already small seasonal latitudinal migrations could be recorded in the δ^13^C values of the baleens.

Assuming little isotopic fractionation of 0.1 ± 1.7‰ or −0.3 ± 1.4‰ (Goericke & Fry, 1994) between δ^13^C_POM_ and copepods (an important sei whale prey, Baumgartner & Fratantoni, 2008) and a Δ^13^C of 2.3‰ for baleens relative to the whale's prey (Borrell et al., 2012), δ^13^C values of baleens should be 2.0–2.4‰ more positive compared to POM. Given a δ^13^C range of −19 to −16‰ for the sei whale baleens, this would translate to average δ^13^C_POM_ of around −21 to −18‰ for their habitats. Such δ^13^C_POM_ values are typical for latitudes north of 50 °S (Goericke & Fry, 1994) and therefore suggest that the investigated sei whales did not enter Antarctic waters. This is in agreement with the observations that sei whales do not migrate as far south as other balaenopterid species and rarely enter into polar waters (Horwood, 1987, 2009; Mizroch et al., 1984) possibly explaining less isotopic variations as in baleens of other whale species.

Similar latitudinal gradients as for δ^13^C exist for δ^15^N in the southern latitudes. For instance, the δ^15^N of nitrate along a latitudinal gradient in the east Pacific decreased by about 3‰ within 20° latitude caused by isotope fractionation due to differential nitrate utilization of phytoplankton (Sigman, Altabet, Mccorkle, Francois, & Fischer, 1999). Such isotopic differences at the baseline of food webs were traced through trophic levels (Jaeger et al., 2010) and in marine sediments (Altabet & Francois, 1994). Presuming that sei whales in the Southern Hemisphere seasonally migrate in a north–south direction and back, latitudinal isotopic differences will cause cyclic δ^15^N and δ^13^C variations in their baleens. Former studies have related δ^15^N and δ^13^C cycles in baleens of southern right whales (*Eubalaena australis*) (Best & Schell, 1996; Hobson & Schell, 1998) and southern humpback whales (Eisenmann et al., 2016) to migration of whales between isotopically distinct areas of food uptake.

Average δ^13^C and δ^15^N ranges of around 2.3‰ and 3.6‰, respectively, as observed in the sei whale baleens, may be translated to a seasonal migration between the Subtropics and Subantarctic zones (Jaeger et al., 2010; Magozzi et al., 2017). However, also offshore/inshore movements could produce isotope variations (Witteveen et al., 2011). Regional baseline data of δ^13^C and δ^15^N could help specifying food sources and possible foraging grounds, but the present data basis of isoscapes for the southeastern Pacific does not allow a clear assignment of the isotope minima to a specific area. Clearly, more baseline data obtained in future studies could improve evaluating migration patterns using southern sei whale baleen isotope records.

### 4.3 Baleen growth rates

Caraveo‐Patiño et al. (2007) found high isotopic variability and irregular annual baleen growth rates in baleen plates of East Pacific gray whales (*Eschrichtius robustus*). In the latter study, the baleen plates of different individuals showed a higher variability in δ^15^N than in δ^13^C, resulting in partly irregular oscillation patterns similar to our study on sei whales. Nevertheless, wavelet analysis provided a tool for inferring baleen growth rates in our study. Wavelet analyses of δ^15^N data suggest mean growth rates of 10.0 − 16.5 cm/year for the investigated sei whales (Figure 3). This corresponds to reported mean baleen growth rates of 15.5 ± 2.2 cm/year in blue whales (*Balaenoptera musculus*) (Busquets‐Vass et al., 2017), of 12.9 cm/year in Pacific common minke whales (*Balaenoptera acutorostrata*) (Mitani, Bando, Takai, & Sakamoto, 2006), of around 20 cm/year in a fin whale (*Balaenoptera physalus*) (Bentaleb et al., 2011), and of 12.0 – 20.0 cm/year in humpback whales (*Megaptera novaeangliae*) (Eisenmann et al., 2016).

## 5 CONCLUSIONS

Seasonal δ^13^C and δ^15^N oscillations were recognizable in all investigated sei whale baleens. Migratory behavior is the most likely explanation for these cyclic patterns. The isotopic composition, especially δ^15^N, of the baleens’ parts formed shortly before death agree with those of potential prey (copepods, euphausiids, amphipods) in Patagonian fjords pointing to this area as a foraging ground. Minimum baleen isotope values do not agree with any Patagonian food source and must have been formed elsewhere, indicating a migratory behavior for all investigated sei whales. The differences in δ^15^N between individuals could result from individual feeding preferences and migratory paths. In particular, individual SE 1 exhibited lower δ^15^N values (8.7–11.6‰) pointing to feeding on a lower trophic level or more offshore than the other individuals. Clearly more regional isotope data of food webs on a latitudinal scale as well as along an offshore/inshore gradient supplemented by tracking studies of Southern Hemisphere sei whales are needed to constrain foraging and calving grounds and to clarify migration routes in future studies.

## CONFLICT OF INTEREST

None declared.

## AUTHOR CONTRIBUTIONS

All authors contributed to the study design and contributed to the manuscript. LR and CM analyzed the data, conducted the research, visualized the results, and wrote a first draft. VH coordinated fieldwork and provided study material.

## Data Availability

Data are available via the database PANGAEA: https://doi.pangaea.de/10.1594/PANGAEA.909235.
